# Patient Perspectives on the Therapeutic Profile of Botulinum Neurotoxin Type A in Spasticity

**DOI:** 10.3389/fneur.2020.00388

**Published:** 2020-05-07

**Authors:** Jorge Jacinto, Pasquale Varriale, Emilie Pain, Andreas Lysandropoulos, Alberto Esquenazi

**Affiliations:** ^1^Centro de Medicina de Reabilitaçãode Alcoitão, Serviço de Reabilitação de Adultos 3, Alcabideche, Portugal; ^2^Carenity, Paris, France; ^3^Ipsen, Cambridge, MA, United States; ^4^MossRehab and Albert Einstein Medical Center, Elkins Park, PA, United States

**Keywords:** botulinum toxin, patient survey, spasticity, waning of effect, patient perspectives

## Abstract

**Background:** Botulinum toxin-A (BoNT-A) injections are first-line treatment for adult spasticity. Prior patient surveys have reported that BoNT-A treatment improves quality of life but that symptoms usually recur before the next injection. We aimed to explore, in-depth, patient perceptions of the impact of spasticity and the waning of BoNT-A therapeutic effects.

**Methods:** An internet-based survey was conducted through Carenity, an online patient community, from May to September 2019 in France, Germany, Italy, UK and USA. Eligible respondents were adult patients with spasticity due to stroke, traumatic brain injury (TBI) or spinal cord injury (SCI) who had ≥2 previous BoNT-A injections.

**Results:** Two hundred and ten respondents (mean 47.2 years) met screening criteria and had their responses analyzed. Overall, 43% of respondents had spasticity due to stroke, 30% due to TBI and 27% due to SCI. The mean [95% CI] injection frequency for spasticity management was 3.6 [3.4–3.7] injections/year. Respondents described the time profile of their response to BoNT-A. The mean reported onset of therapeutic effect was 12.9 [12.1–13.7] days and the mean time to peak effect was 5.0 [4.7–5.4] weeks. Symptom re-emergence between injections was common (83%); the time from injection to symptom re-emergence was 89.4 [86.3–92.4] days. Muscle spasms usually re-emerge first (64%), followed by muscle stiffness or rigidity (40%), and limb pain (20%). Over half (52%) of respondents said they had lost their self-confidence, 46% experienced depression and 41% experienced a lack of sleep due to their spasticity symptoms in the past 12 months. Following a report of symptom re-emergence, the most common management approaches were to add adjunctive treatments (36%), increase the BoNT-A dose (28%), and wait for the next injection (27%). Seventy two percentage of respondents said they would like a longer lasting BoNT-A treatment.

**Conclusions:** Patients with spasticity can expect a characteristic profile of BoNT-A effects, namely time lag to onset and peak effect followed by a gradual decline in the symptomatic benefits. Symptom re-emergence is common and has significant impact on quality of life. Greater patient/clinician awareness of this therapeutic profile should lead to better level of overall satisfaction with treatment, informed therapeutic discussions and treatment schedule planning.

## Introduction

Spasticity is caused by an upper motor neuron (UMN) lesion leading to intermittent or sustained involuntary activation of muscles (1), and is a common problem that interferes with function in people recovering from a stroke, traumatic brain injury (TBI) or spinal cord injury (SCI) (2). The pattern of spasticity experienced varies enormously depending on the type, location, size and chronicity of the UMN lesion. Overactive muscle activation (neurogenic component) and stiffening and shortening of the muscle and other soft tissues (rheologic component) are the two main contributory factors to movement resistance in the limbs after UMN damage (3). No two patients present in the same way, and this heterogeneity makes the identification, classification and rehabilitation process complex and challenging, requiring tailoring to each individual (4). This process is often crucial to adequately manage spasticity (5, 6).

Botulinum toxin type A (BoNT-A) is a mainstay pharmacological treatment for the management of spasticity (3, 7). It exerts its effects by binding presynaptically to high-affinity recognition sites on the cholinergic nerve terminals, hence inhibiting the release of acetylcholine, causing temporary neuromuscular blockade with muscle relaxation. The effects of BoNT-A are not permanent, and neurotransmission gradually resumes as the neuromuscular junction recovers (8).

The translation from BoNT-A pharmacological effects at the neuromuscular junction to the lived patient experience varies, but clinical studies have shown a significant reduction in muscle tone (vs. baseline and placebo) as early as 1 week post intramuscular injection, with peak effects at 4–6 weeks and waning of effect thereafter (9, 10). While it is clear that patient satisfaction with treatment is lower at end of cycle (before next injection) than at peak effect (11), patient perceptions of treatment efficacy over a full treatment cycle, the personal impact of symptom re-emergence and the patient-related triggers for reinjection have not been fully studied to date. The aim of this online survey was to explore patient perceptions of the impact of spasticity and how they experience the waning of BoNT-A therapeutic effects.

## Methods

### Survey Design

This international online survey was conducted between May 15, 2019 and September 16, 2019. The structure and contents of the survey were designed collaboratively between the authors and the online patient community Carenity (Paris, France). Questions were designed to document and explore sample characteristics (demographics and medical history), current treatment for spasticity, BoNT injection experiences, experiences of symptom re-emergence, impact of symptom re-emergence on quality of life and physician-patient and caregiver communication about symptom re-emergence. Before survey deployment, all questions were preliminarily tested with two individuals living with spasticity and currently receiving BoNT-A treatment for their symptoms (one in the US and one in Europe) and refined to improve ease of understanding and relevance to the patients.

Most questions were multiple choice with some allowing input of free text. Severity of symptoms and impact on quality of life at different time points were rated on analog scales, ranging from 0 (no impact on quality of life) to 10 (very strong symptoms/very strong impact on quality of life). In order to assess the impact of symptom re-emergence on quality of life, we asked respondents to consider their “ability to move around,” “perform daily tasks,” “self-confidence,” “sleep” and “fatigue and relationships with family and friends.” We also tested the respondents ability to focus and understand the questionnaire format by including three times the same extra question where they were asked to check every option with a number value higher than 5.

The survey was designed to be self-completed by the respondents. It was first designed in English, and then translated in French, German and Italian. The survey was hosted online and included 40 demographic and disease related questions (Supplementary Material). The survey was designed to take ~20–25 min to complete, although there was no set time limit for completion.

### Recruitment and Survey Participants

The study was conducted in compliance with relevant codes of conduct and data protection legislation. Clinical Research Ethics Committee or Independent Review Board approval was not required for this exploratory patient satisfaction survey. All participants provided informed consent to participate. They were made aware that the research was sponsored by a pharmaceutical company interested in the treatment for spasticity.

People living with spasticity due to stroke, TBI or SCI, either as a patient or as a caregiver, were invited to participate in the survey via Carenity, an online patient community for both patients and caregivers of patients living with chronic disease who also hosted the survey on their country websites. In addition, the Stroke Alliance for Europe (SAFE) and the Brain Injury Association of America (BIAA) also shared the survey with their members via email and/or newsletters and social media. Eligible respondents were adult (≥18 years old) persons (or caregivers of persons meeting these criteria) undergoing treatment with BoNT-A (≥2 injections) and living in France, Germany, Italy, the United Kingdom or the United States of America. Respondents who had stopped BoNT-A treatment in the last 12 months were also eligible to participate.

### Data Analysis

Descriptive statistics were used to summarize all survey data collected in this study. To better understand the experience of BoNT-A effects, we looked at the time to onset, peak and waning of effect overall and stratified by etiology.

## Results

### Sample Characteristics

Taken overall, there were 721 unique respondents to the online survey. Of these, 210 respondents met the survey screening criteria and were included in the analyses. The main reasons for screen failure were etiology of spasticity (e.g., due to multiple sclerosis) and not receiving BoNT-A treatment. Nine respondents were caregivers of individuals living with spasticity who answered questions about BoNT-A experiences for the patient. The mean [95% CI] respondent age was 47.2 [45.9–48.5] years old and 53% of respondents were male (Table 1). Overall, 43% of respondents had spasticity due to stroke, 30% due to TBI, and 27% due to SCI. The mean [95% CI] age of onset of the neurological event was 42.7 [41.3–44.2] years old and the mean time since event was 4.6 [3.8–5.4] years. Respondents with post-stroke spasticity were older at the time of event than those with TBI and SCI (mean age of 45.3 vs. 40.6 and 40.8 years old, respectively). This age distribution indicates a younger population with stroke responded to the survey compared to incidence data (12). Across the etiologies, most respondents were employed (full or part-time).

**Table 1:** Respondent characteristics.

**Characteristic**	**Statistic *N* = 210**
Country; n (%)	
France	29 (14)
Germany	26 (12)
Italy	30 (14)
United Kingdom	20 (10)
United States of America	105 (50)
Sex; n (%)	
Female	99 (47)
Male	111 (53)
Employment status; n (%)	*N* = 207^*^
Full time	76 (38)
Part time due to spasticity	72 (35)
Part time (not due to spasticity)	10 (5)
Do not work due to spasticity	32 (16)
Do not work (not due to spasticity)	15 (8)
Student	2 (1)
Age (years); mean [95% CI]	47.2 [45.9, 48.5]
Age category; n (%)	
<40 years old	39 (19)
41–50 years old	110 (52)
51–60 years old	48 (23)
>60 years old	13 (6)
Age at time of event (years); mean [95% CI]	42.7 [41.3, 44.2]
Etiology; mean [95% CI]	
Stroke	45.3 [43.3, 47.4]
TBI	40.6 [37.5, 43.7]
SCI	40.8 [38.6, 43.0]
Time since event (years); mean [95% CI]	4.6 [3.8, 5.4]
Time category; n (%)	
<2 years	44 (21)
2–5 years	118 (57)
6–10 years	26 (12)
>10 years	19 (9)
Do not remember	3 (1)
Symptoms experienced in past year; n (%)	
Muscle stiffness/rigidity	148 (70)
Muscle spasms	132 (63)
Muscle pain	108 (51)
Unwanted movements of the affected limb	86 (41)
Difficulties moving my leg, falling, tripping, loss of balance	104 (50)
Difficulties moving my arm/hand, extending my arm, opening my hand	82 (39)
Currently receiving BoNT-A; n (%)	203 (97)
AbobotulinumtoxinA (Dysport)	41 (20)
IncobotulinumtoxinA (Xeomin)	44 (21)
OnabotulinumtoxinA (Botox)	112 (53)
BoNT-A product unknown	*N* = 6 (3)
No (stopped in past 12 months)	7 (3)
Time since first BoNT-A injection	
<2 years	102 (48)
2–5 years	88 (42)
>5 years	12 (6)
Do not remember	8 (4)
Injected limbs; n (%)	
1 Upper limb only	53 (25)
Both upper limbs	37 (18)
1 Lower limb only	42 (20)
Both lower limbs	42 (20)
One side (upper and lower limbs)	31 (15)
Diagonal (upper and lower limbs)	4 (2)
≥2 Limbs	1 (0)

* *Employment status for respondents > 65 years old was not analyzed*.

The vast majority (97%) of respondents were currently receiving BoNT-A therapy. Of the nine caregivers, 5 said they answered for the patient and 4 said they answered with the patient; five caregivers were male and 4 were female and the mean age of the caregiver was 59.2 years old. Most of their respective patients had stroke (7 of 9) and 1 each had TBI and SCI.

### Current Treatment for Spasticity

As per eligibility criteria, most (97%) respondents were currently receiving BoNT-A therapy (mean [95% CI] duration of 2.2 [1.9, 2.5] years). Two respondents had previously received BoNT-A injections in the prior 12 months but were not currently receiving any treatment (of any type). Over half (53%) said they received onabotulinumtoxinA (Botox®), 21% received incobotulinumtoxinA (Xeomin®), 20% received abobotulinumtoxinA (Dysport®), and 6% did not know the name of the product. Overall, 53% were treated with BoNT-A in more than one limb, 43% were only receiving injections into the upper limbs and 40% were only receiving injections into the lower limbs, 17% were treated in both upper and lower limbs at the same time.

**Figure 1:**
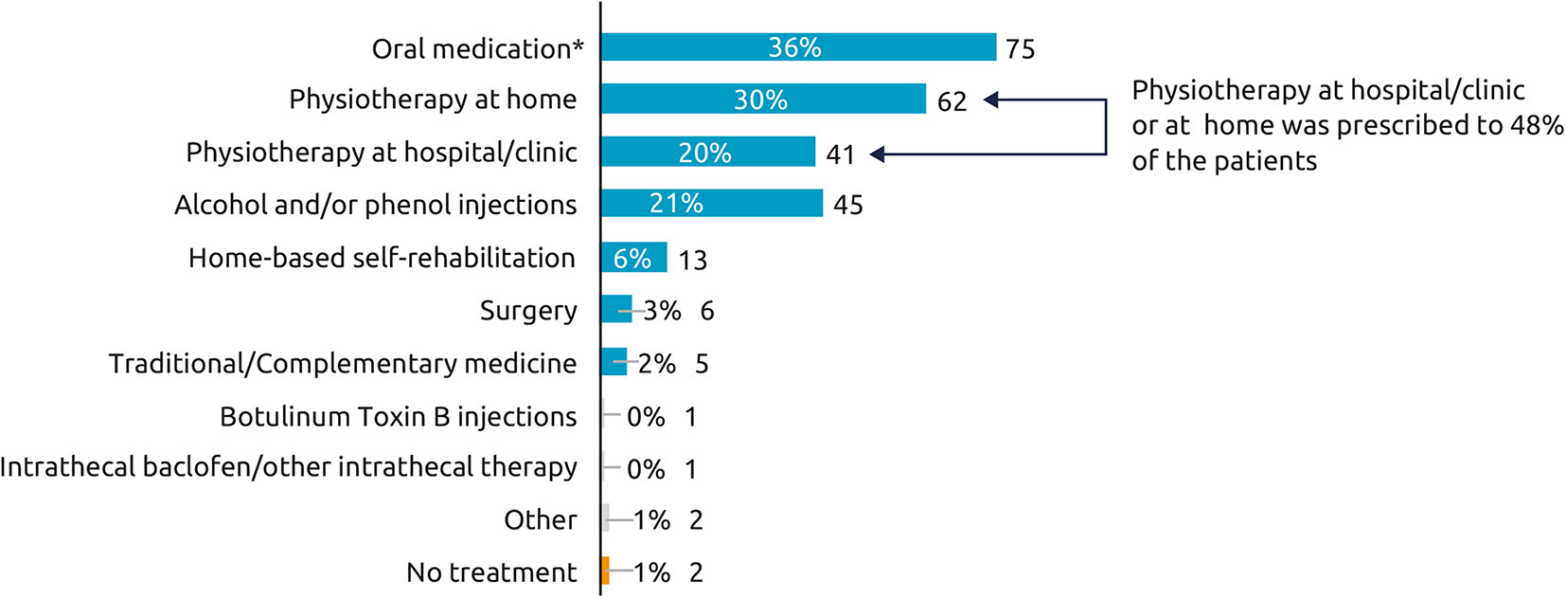
Current therapy for spasticity (other than BoNT-A). Which treatments are you currently receiving for your spasticity? (*N* = 210), *Oral medications such as muscle relaxant or baclofen. Overall, 97% respondents were currently receiving BoNT-A and 3% had stopped within the year prior to the survey.

Across the etiologies studied, 61% of respondents started their treatment with BoNT-A injections within 2 years of the neurological event. The mean [95% CI] time between event and first injection was 1.8 [1.2, 2.3] years for post-stroke, 2.7 [0.7, 4.6] years for TBI, and 2.0 [1.0, 3.0] years for SCI. As shown in Figure 1, most respondents (70%) were currently treated with ≥1 treatment intervention (i.e., BoNT-A plus another therapeutic intervention). Rates of concomitant oral medications (e.g., muscle relaxant or baclofen), were substantially higher in respondents with spasticity due to SCI (58%) than stroke (30%) and TBI (24%). Conversely, post-stroke respondents were somewhat more likely to report additional chemodenervation with alcohol or phenol (23%) than those with spasticity due to TBI (17%) or SCI (14%). Rates of physiotherapy (at home or clinic) were also higher in respondents with spasticity due to stroke (56%) compared with those with spasticity due to SCI (47%) and TBI (38%).

### Botulinum Neurotoxin Type A Injection Experiences

The mean injection frequency for spasticity was 3.6 [3.4, 3.7] injections per year, and was similar across the etiologies. Most respondents said they received 3 (*n* = 57, 27%), 4 (*n* = 99, 48%), or 5 (*n* = 20, 10%) injections per year. Only 3 respondents (1%) received 6 injections per year and 12% received ≤2 injections per year. Accordingly, 72% of respondents said they had their last 2 sessions within 3–4 months of each other, while 7% reported having injections with intervals of <3 months and 20% reported injection intervals of ≥4 months. Almost half of the respondents (*n* = 103, 49%) said their injection interval was always the same, and of these, most (79%) said the schedules were well-adapted to their needs. Likewise, of the 90 (43%) respondents whose injection scheduling was flexible depending on their symptoms, 89% reported satisfaction with the injection schedules. Thirteen (6%) respondents said their sessions depended on their availability and another four (2%) said their sessions depended on physician availability.

**Figure 2:**
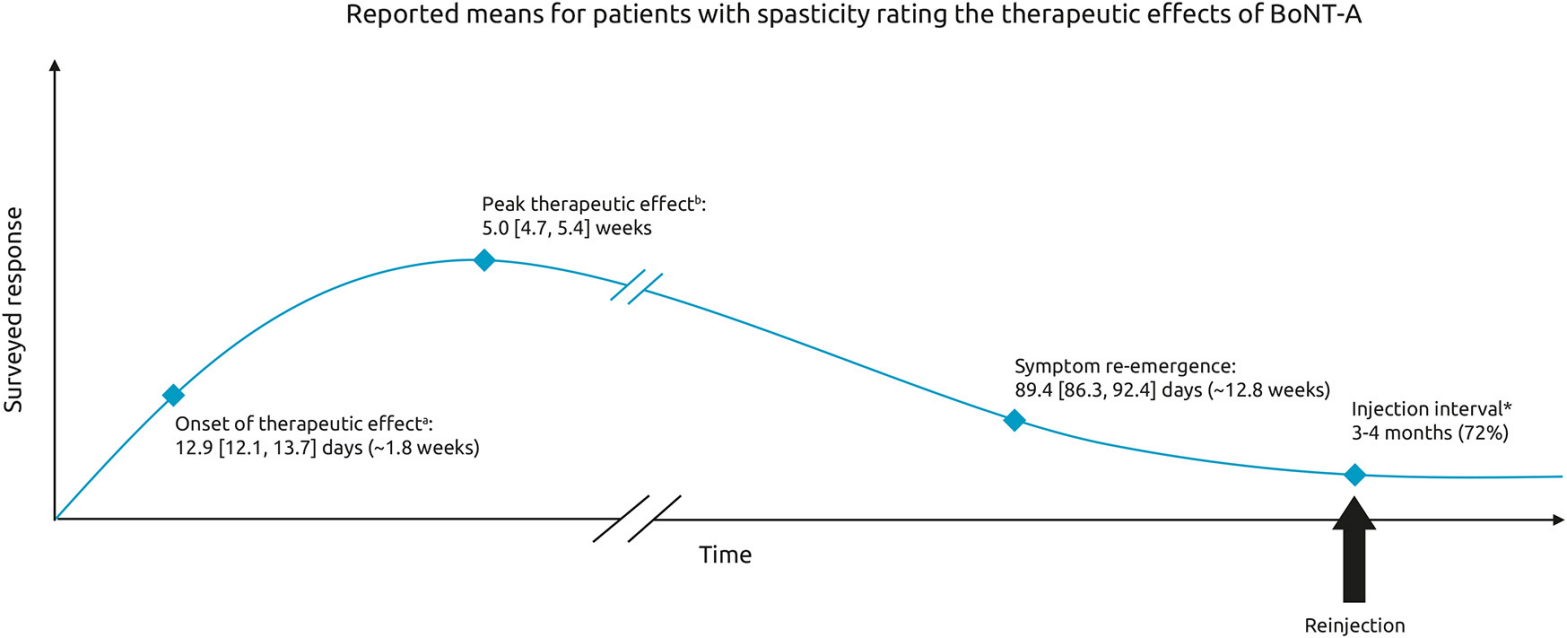
Therapeutic effects of BoNT-A. Schematic representing the mean [95% CI] time to onset, peak therapeutic effect and time to symptom re-emergence. Respondents were asked: On average, how many days or weeks after your BoNT-A injections do you experience. ^a^The first effect of the treatment on your spasticity ^b^the maximum effects of the treatment on your spasticity (in days or weeks). In general, how long after your BoNT- A injections do your pre-existing symptoms begin to reappear. *Respondents were also asked to indicate the time between the last two sessions of Botulinum Toxin A [multichoice question].

When asked to describe the onset of therapeutic effect, 21% of respondents said they noticed effects within 9 days, 50% said they noticed effects within 10–14 days, and 20% said it took >15 days to notice the first effects of BoNT-A treatment on their spasticity. The mean [95%CI] onset reported was 12.9 [12.1, 13.7] days and was relatively consistent across all etiologies (12.0–14.0 days) (Figure 2). The mean time to peak effect was 5.0 [4.7, 5.4] weeks, however reports were variable, with 36% of respondents reporting that they typically experience peak effect within a month, 27% within 1–2 months and 9% reporting it takes longer than 2 months to reach peak effect. The remaining 28% of respondents were unable to determine the time to peak effect (answered don't know). Across the etiologies, most respondents reported onset of effect within 2 weeks. However, respondents who had a SCI reported that they experience the maximum effects of BoNT-A injections somewhat later than those with spasticity due to stroke or TBI (mean [95% CI] of 5.7 [5.0–6.3] weeks vs. 4.8 [4.3, 5.3] weeks and 4.8 [4.1, 5.5] weeks, respectively).

### Experiences of Symptom Re-emergence

Symptom re-emergence between injections was common, with 83% of respondents saying they noticed their pre-existing symptoms reappearing between 2 injection sessions, and this was consistent across all etiologies (82–84%). The mean time to re-emergence of pre-existing symptoms was 89.4 [86.3, 92.4] days after BoNT-A injection, with 6% reporting symptom re-emergence within 2 months, 53% reporting within 2–3 months and 28% reporting symptom re-emergence only after >3 months. Overall, 22 respondents (13%) could not define the time to re-emergence of pre-existing symptoms. Of note, respondents receiving BoNT-A and concomitant oral medications experienced symptom re-emergence more frequently than those who only received BoNT-A injections and those who also received physiotherapy (89 vs. 81 and 79%, respectively).

**Figure 3:**
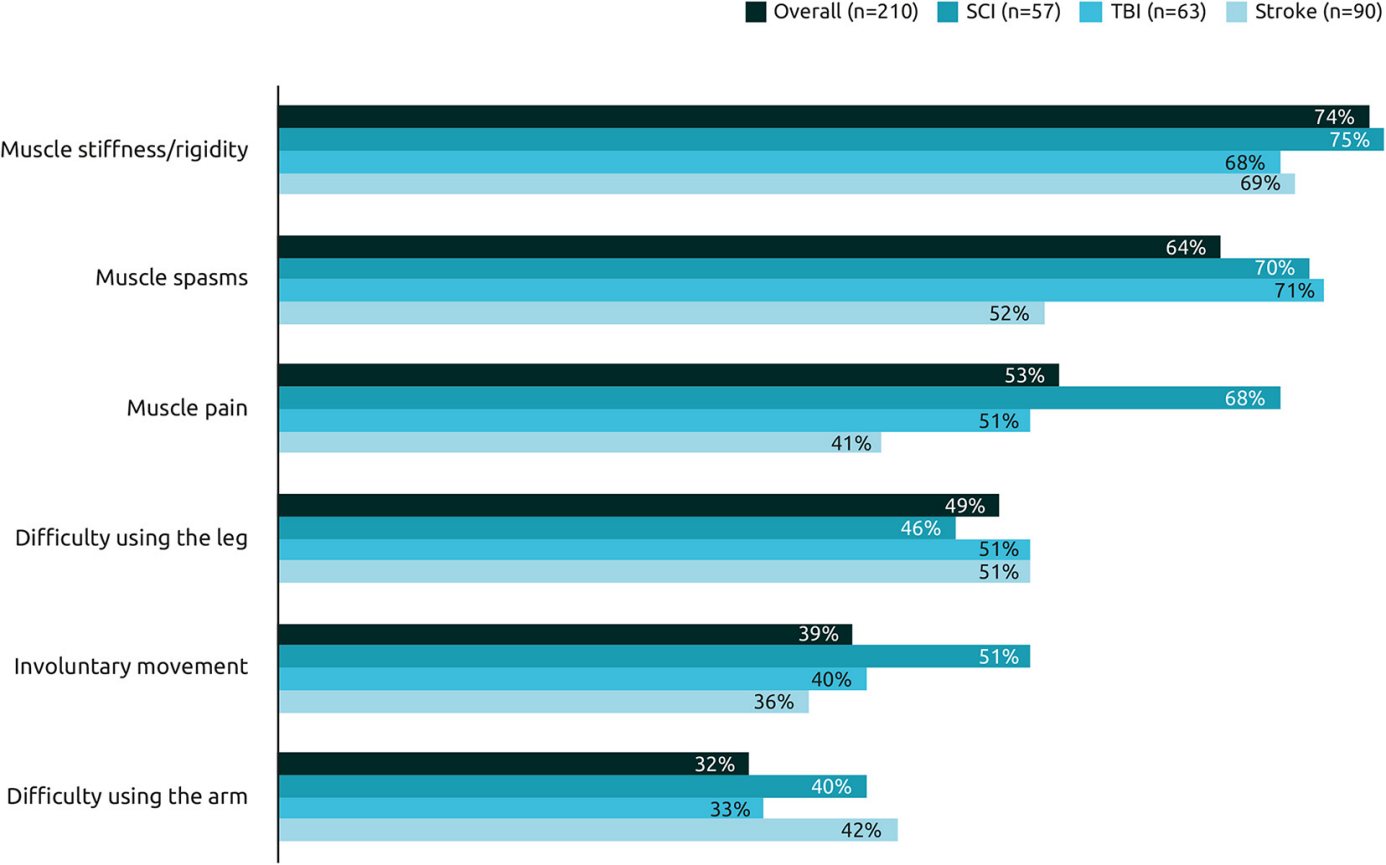
Frequency of re-emergent symptoms by etiology. Among the following pre-existing symptoms, could you select the ones which reappear between two sessions of BoNT-A injections in their order of reappearance?

On average, respondents experienced re-emergence of 3.1 symptoms between two sessions of BoNT-A injections. The most common re-emergent symptoms were muscle stiffness/rigidity (74%), followed by muscle spasms (64%), and muscle pain (53%) (Figure 3). Symptoms were generally similar across the etiologies, however respondents with spasticity due to stroke were less likely to report muscle spasms or pain than those with spasticity due to TBI or SCI.

**Figure 4:**
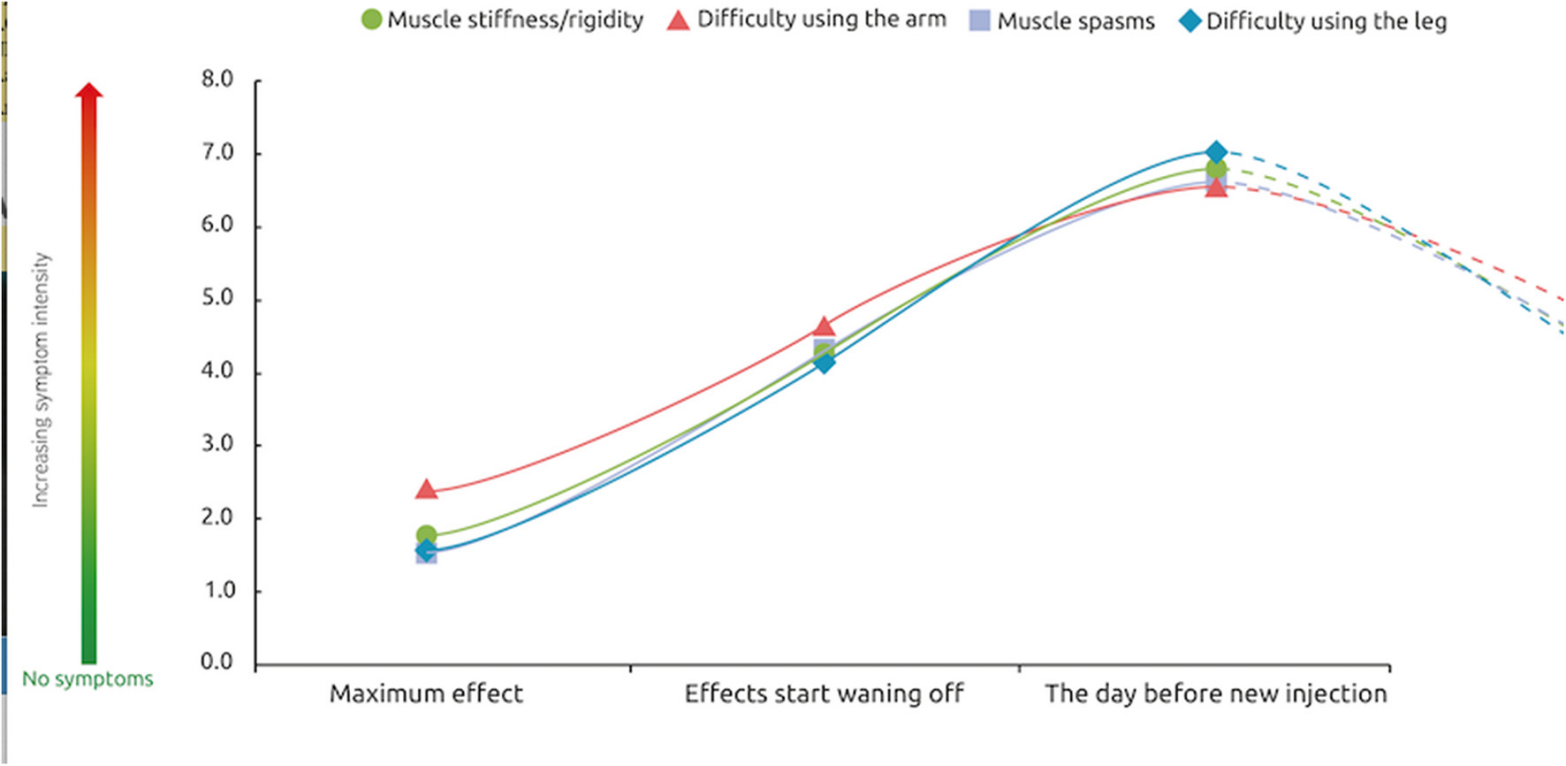
Severity of symptoms over a typical BoNT-A injection cycle. At these 3 different points of treatment [peak effect, waning of effect, just prior to next injection], how would you rate the intensity of your symptoms? *n* = 174 respondents whose symptoms reappear between two sessions of injections.

Respondents were asked to rate the intensity of their symptoms at peak effect, at waning of effect and 1 day prior to their next injection. Treatment was not reported to completely abolish symptoms, even at peak effect. However, symptom severity was lowest at the peak of treatment effects (score of ~1.5 out of a maximum 10), increasing as the effects of treatment start wearing off (around 4 of 10) and was strongest 1 day before the next session (7 out of 10) (Figure 4). This “rollercoaster” of varying symptom intensity was similar across the etiologies.

### Impact of Symptom Re-emergence on Daily Life

**Figure 5:**
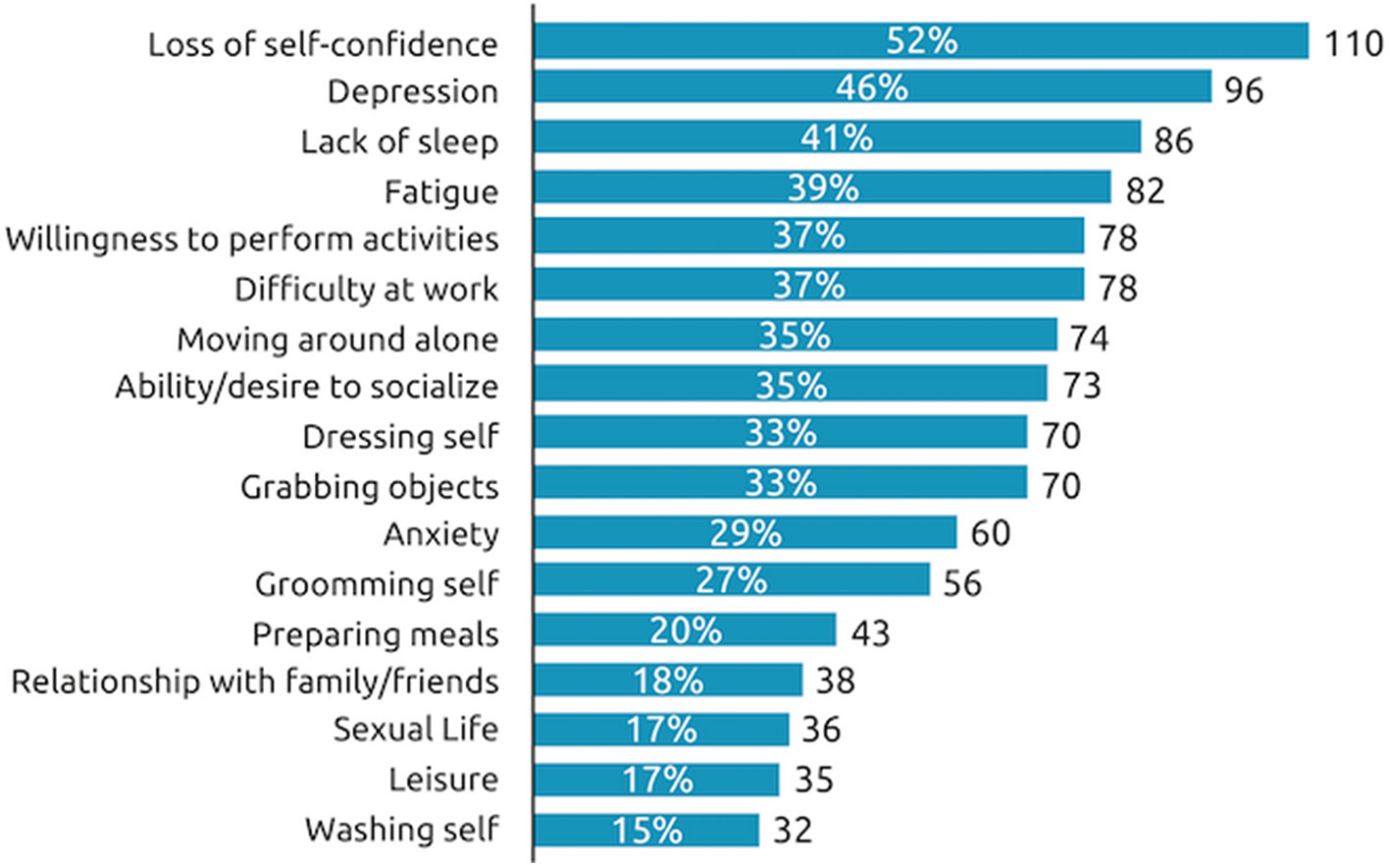
Impact of symptom re-emergence in past year. During the past 12 months, at the worst time, which of the following situations have you experienced as a consequence of your spasticity? *n* = 210 respondents.

When considering the impact of their spasticity over the past 12 months, 52% of all respondents said they had lost their self-confidence, 46% said they had experienced depression and 41% said they had experienced a lack of sleep due to their spasticity symptoms in the past 12 months (Figure 5). Respondents with SCI tend to be impacted in more aspects of their daily life than those with stroke or TBI, especially in the quality of their sleep (53% respondents with SCI reported sleep impact vs. 37% of respondents with stroke and 37% with TBI) and their ability to move around independently (46% respondents with SCI reported impact on independent movement vs. 32% of respondents with stroke and 30% with TBI).

**Figure 6:**
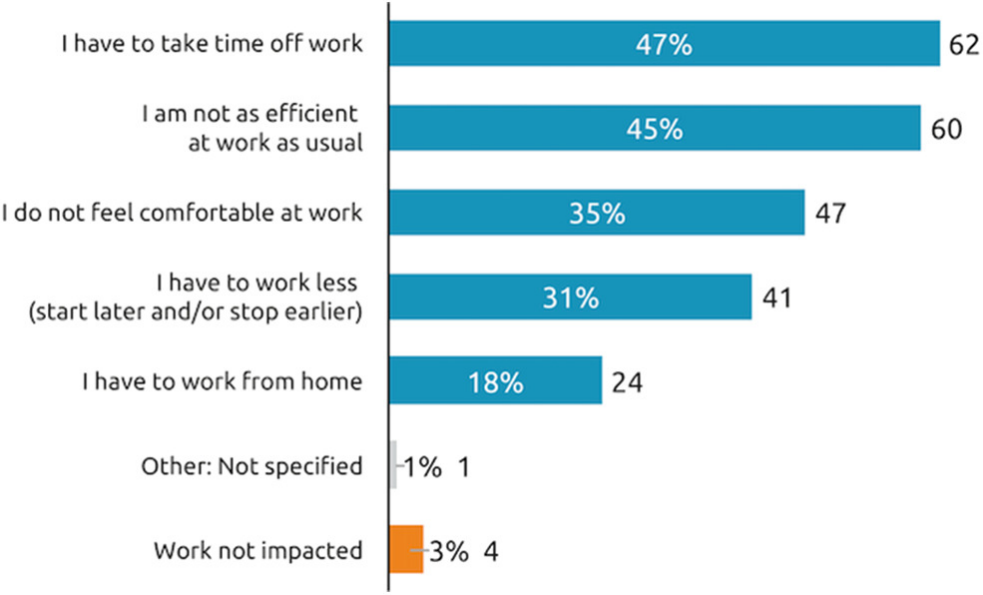
Impact of symptom re-emergence on professional life (working patients). How does the reappearance of your spasticity pre-existing symptoms between two sessions of BoNT-A injections affect your work?, *n* = 133 respondents who are currently working and whose symptoms reappear between two sessions.

Rates of employment (full or part time or student) for working age respondents were similar across the etiologies. Taken overall, when people who were still working (*N* = 133) were questioned further about the impact of symptom re-emergence on their professional life, 47% said have to take time off work and 45% said they are not as efficient at work as usual (Figure 6).

**Figure 7:**
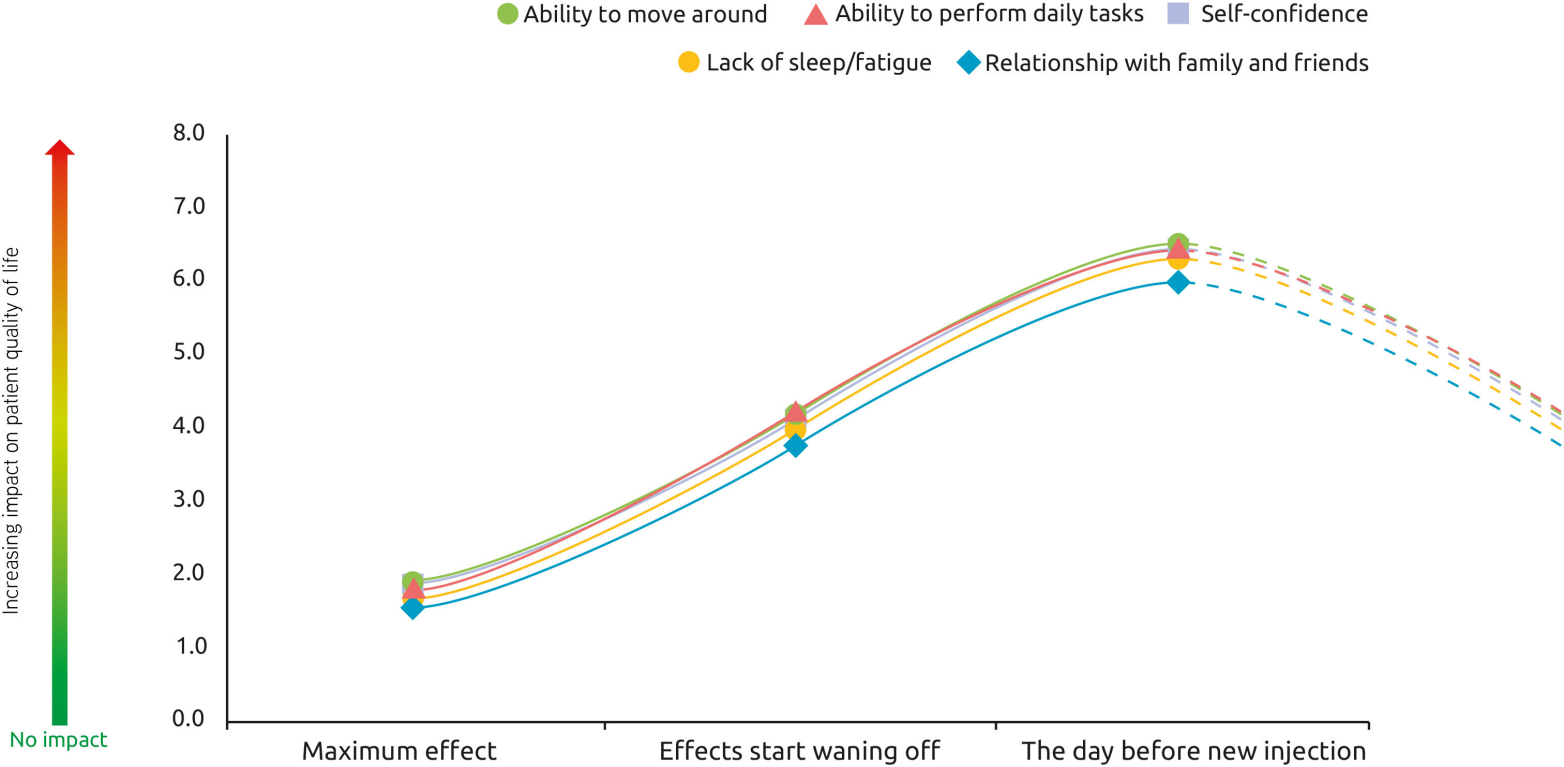
Impact of spasticity on patient quality of life over a typical BoNT-A injection cycle. At these 3 different points of treatment [peak effect, waning of effect, just prior to next injection], how would you rate the impact of spasticity on your quality of life?, *n* = 174 respondents whose symptoms reappear between two sessions of injections.

The impact of recurring symptoms on quality of life follows the same pattern across quality of life domains. As expected, the impact on quality of life was rated as smallest at the peak of treatment effects, with increasing impact as the effects of treatment start wearing off until the next injection session (Figure 7).

### Physician-Patient Communication About Symptom Re-emergence

Most respondents (94%) said they had discussed the potential for symptom re-emergence between injections with their doctor. When asked whether they report symptom re-emergence to their physician, most (92%) said they inform their physician. The majority (66%) said they report their symptom re-emergence immediately, regardless of symptom intensity while 15% only reported symptom re-emergence if the symptoms were severe. Overall, 8% of respondents (*n* = 12) said they do not inform their doctor. Following a report of symptom re-emergence, the most common management approaches were to add adjunctive treatment (36%), increase the dose of BoNT-A (28%) and to wait for the next injection (27%). Another 26% reported they went back to their physician for an earlier than planned session.

**Figure 8:**
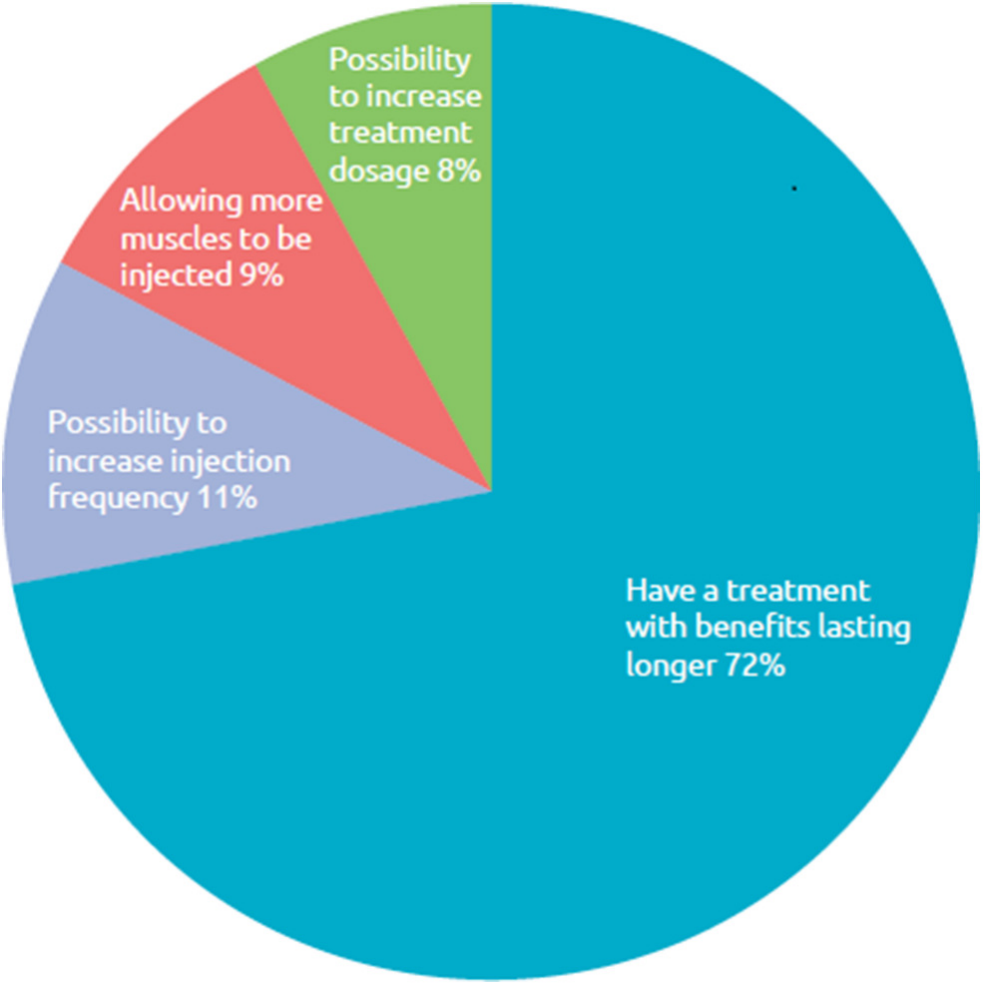
Respondent wish for improved BoNT-A treatment. What improvements with your BoNT-A treatment do you want in order to avoid reappearance of symptoms between sessions of injections?, *n* = 169 respondents whose symptoms reappear between two sessions of injections.

Of the 12 respondents who said they do not report symptom re-emergence, 42% (*n* = 5) said they did not think their doctor could do anything more about it. Finally, when asked what improvements with BoNT-A treatment they wished for in order to avoid symptom re-emergence between sessions, most (72%) said they would like a longer lasting BoNT-A treatment, while 11% considered shorter re-injection intervals (Figure 8).

## Discussion

To our knowledge this is one of the largest electronic surveys to provide in-depth evaluation of how patients living with spasticity experience the therapeutic effects of BoNT-A treatment and the impact on quality of life of symptom re-emergence between injection sessions. Survey findings showed that patients living with spasticity typically experience a re-emergence of their muscle stiffness, spasms, and pain between injection sessions, with important impacts on their daily activities and quality of life.

Although clinical studies have shown significant reductions in hypertonia (as assessed by Modified Ashworth Scale scores) as early as 1 week (9) our findings indicate that most patients only appreciate the benefits of treatment after at least 12–13 days. While the time to onset was fairly consistent across etiologies, there was considerable variation in the reported time to peak effects. This was especially apparent in respondents with spasticity due to SCI who reported reaching peak effects about a week later than those with spasticity due to stroke or TBI. Differences may be related to treatment goals or the equivalent per muscle dose used in patients with SCI who often have more muscles involved requiring treatment. However, taken on average, patient perception matched established clinical data, which suggests that it takes 4–6 weeks for BoNT-A treatment to reach peak efficacy. Of note, respondents with spasticity due to SCI were more likely to report concomitant treatment with oral therapies, indicating a need for additional control of their more generalized spasticity presentation. In addition, use of oral medications may be lower in stroke and TBI survivors because of cognitive deficits that may be worsened by the central effects exerted by these medications (13, 14).

Symptom re-emergence between injections was the norm with most respondents (83%) reporting the reappearance of symptoms between 2 injections. Among them, waning of effects were reported to start occurring about 12–13 weeks after the injection. The intensity of symptoms and their subsequent impact on quality of life “fluctuated” in line with the time course of BoNT-A effects. It is pertinent to note that respondents reported that they still experience a relatively low level of symptom intensity (scoring between 1 and 2 out of 10), even at peak BoNT-A effect. Symptoms were generally reported as being of mild to moderate intensity (scores of 4 out of 10) at the time they noticed symptom re-emergence and were reported to be more severe (scores of 6–7 out of 10) 1 day before the next injection. A limitation of the survey is that we don't know if patients equated a score of 10 “very strong symptoms” with their worst severity experienced. The impact of recurring symptoms on quality of life followed the same pattern across all domains evaluated. The symptoms of spasticity similarly affected the respondents' ability to move around and perform daily tasks, as well as affecting their self-confidence, relationships and quality of sleep.

With a mean age of 47.2 years, our survey sample was noticeably younger than other surveys of spasticity (11, 15) and epidemiological data—perhaps as a consequence—the proportion reporting to be working (78%) was higher than expected. The impact on those respondents who work was striking: 97% of working patients reported that their work was affected by symptom recurrence; 47% said they have to take time off work when the symptoms re-emerge; and 45% reported a loss of efficiency. It should be noted that our data highlight the fact that a considerable number of people living with spasticity can, and do, find employment. Such data emphasizes the need for more research in this area particularly among patients under the age of 50 years of age. Aside from work done in adult cerebral palsy (16) and multiple sclerosis (16), most research to date has focused on the impact on employment status of those caring for a person living with severe spasticity (17, 18) rather than on the affected persons themselves.

Our findings offer some important points for physician–patient discussion during treatment initiation and goal setting, monitoring and achievement. The data show that, from the patients' perspective, the effects of BoNT-A are not immediate, take time to reach their full potency, and do not always last throughout the time interval between two sessions. Given the high expectations many patients have of BoNT-A therapy (15), it is important that they understand the realistic objectives and the limitations of their treatment, and the probable time course of symptom relief. Injectors can only adjust the treatment regimen if they have adequate information at hand, and this will often depend on the patient being able to communicate how and when they re-experience their symptoms, as well as what is the maximum level of severity they consider acceptable just before the next injection. Our results show that many patients are able to describe the time course of effects in a consistent way. In this survey only 8% of respondents did not inform their doctor of the reappearance of their symptoms between two sessions of injections. This number was lower than we expected, and may reflect the population sample which is relatively young, with good cognitive function, and a proactive approach to disease management (as evidenced by joining a social media group for people living with chronic conditions). Patients with cognitive dysfunction or a lesser functional expectation to their condition may require some prompting questions to tease out these points. Indeed, 13% of respondents in this survey could not readily define the usual time taken to symptom reemergence. As such, it may also be helpful to develop educational visual tools (app based or diaries) explaining what to expect from BoNT-A treatment and when, allowing patients or their caregivers to record their experience, including waning of effect.

Most respondents reported being generally satisfied with their current injection schedules. However, when asked what they would like as an improvement to their BoNT-A therapy, most indicated they would prefer a regimen longer lasting with less frequent injections. This is in direct contrast to a prior survey where patients indicated they would prefer shorter than the standard 12 week intervals to match their duration of efficacy (11). The reasons for these discrepancies are unclear but may, for example, reflect the way the questions were framed and a lack of patient awareness of the possibilities for adjusting treatment in other ways. There is a clear BoNT-A dose relationship for duration of effect, and in cases of shorter than desired intervals, clinicians can consider several ways of improving the regimen such as increasing the dose of BoNT-A delivered (total doses and/or doses to specific muscles) or reconsidering which muscles should be injected. In addition, recent real-world data from the ULIS-3 observational study in adult upper limb spasticity suggest that there may be differences between BoNT-As in terms of the duration of symptom relief between injections (19).

Limitations of this study are those inherent to patient surveys, which are based on the patient's own understanding of their condition and not cross-checked with clinical information. There was no verification of the spasticity diagnosis or verified information on important treatment parameters (e.g., BoNT-A doses used, treatment goals, injection intervals), which can influence patient satisfaction and the duration of effect. We also did not consider the treatment setting or region of the world and how this may affect the patients' experiences. Another limitation is the recruitment method, in which only those patients with the ability and competency to use the Internet would be likely to respond to the survey (likely excluding post-stroke and TBI patients with more significant cognitive dysfunction). As mentioned earlier, the Carenity community likely engages younger, female patients, which may account for the younger sample than in other reported surveys (20). This skew to a younger, more cognitively-able sample likely explains the surprisingly high proportion of working patients. While a larger sample size would have been desirable, responses from >200 eligible patients were considered adequate to characterize the patient experience of treatment with BoNT-A therapy. In our analyses, we chose to report the combined analyses of patients and caregivers and a larger caregiver sample would be required before trying to make any comparisons of the findings.

## Conclusions

In summary, this large BoNT-A treatment survey found that patients living with spasticity should expect a characteristic profile of BoNT-A effects, namely time lag to onset and peak effect followed by a gradual decline in the perceived symptomatic benefits. Symptom re-emergence is common and has significant impact on daily activities and quality of life. Accordingly, patients say they would prefer treatments with a longer duration of effect. Greater patient awareness of this therapeutic profile should lead to better education, informed therapeutic discussions, optimization of dosing, and schedule planning.

## Data Availability Statement

Ipsen will share all individual participant data that underlie the results reported in this article with qualified researchers who provide a valid research question. Proposals should be submitted to DataSharing@Ipsen.com and will be assessed by a scientific review board. Data are available beginning 6 months and ending 5 years after publication.

## Ethics Statement

Ethical review and approval was not required for the study on human participants in accordance with the local legislation and institutional requirements. The patients/participants provided their written informed consent to participate in this study.

## Author Contributions

All authors were involved in survey development, data review, analysis, and contributed to the interpretation of results and approved the final version of the article. PV and EP were involved in survey conduct and were responsible for data analysis.

## Conflict of Interest

This study was funded by Ipsen Pharma. One author employed by Ipsen (AL) was involved in the survey design; in interpretation of the data; and in review, approval of, and decision to submit the manuscript. The funder had no other role in study conduct or preparation of this report. AE reports serving as a scientific advisor for Ipsen and Allergan and receiving research support from Ipsen, Allergan and Merz. JJ has received honoraria for lecturing, scientific advisory, peer training from Ipsen, Allergan and Merz. PV and EP are employed by Carenity, a company that was contracted to perform the survey reported.
