# Corrigendum: Patient Perspectives on the Therapeutic Profile of Botulinum Neurotoxin Type A in Spasticity

**DOI:** 10.3389/fneur.2020.629181

**Published:** 2020-12-17

**Authors:** Jorge Jacinto, Pasquale Varriale, Emilie Pain, Andreas Lysandropoulos, Alberto Esquenazi

**Affiliations:** ^1^Centro de Medicina de Reabilitaçãode Alcoitão, Serviço de Reabilitação de Adultos 3, Alcabideche, Portugal; ^2^Carenity, Paris, France; ^3^Ipsen, Cambridge, MA, United States; ^4^MossRehab and Albert Einstein Medical Center, Elkins Park, PA, United States

**Keywords:** botulinum toxin, patient survey, spasticity, waning of effect, patient perspectives

In the original article, there were three data errors, due to the raw data being read from the wrong columns of the data tables.

Corrections have been made to the following sections: *Abstract, Results Summary*; *Results, Sample Characteristics, Paragraph 1*; *Results, Experiences of Symptom Re-emergence, Paragraph 1*; *Results, Physician-Patient Communication About Symptom Re-emergence, Paragraph 1*; and *Discussion, Paragraph 5*.

The corrected paragraphs are presented below.

***Abstract, Results Summary***

**Results:** Two hundred and ten respondents (mean 47.2 years) met screening criteria and had their responses analyzed. Overall, 43% of respondents had spasticity due to stroke, 30% due to TBI and 27% due to SCI. The mean [95% CI] injection frequency for spasticity management was 3.6 [3.4–3.7] injections/year. Respondents described the time profile of their response to BoNT-A. The mean reported onset of therapeutic effect was 12.9 [12.1–13.7] days and the mean time to peak effect was 5.0 [4.7–5.4] weeks. Symptom re-emergence between injections was common (83%); the time from injection to symptom re-emergence was 89.4 [86.3–92.4] days. Muscle spasms usually re-emerge first (64%), followed by muscle stiffness or rigidity (40%), and limb pain (20%). Over half (52%) of respondents said they had lost their self-confidence, 46% experienced depression and 41% experienced a lack of sleep due to their spasticity symptoms in the past 12 months. Following a report of symptom re-emergence, the most common management approaches were to add adjunctive treatments (36%), increase the BoNT-A dose (28%), and wait for the next injection (27%). Seventy two percentage of respondents said they would like a longer lasting BoNT-A treatment.

***Results, Sample Characteristics, Paragraph 1***

Taken overall, there were 721 unique respondents to the online survey. Of these, 210 respondents met the survey screening criteria and were included in the analyses. The main reasons for screen failure were etiology of spasticity (e.g., due to multiple sclerosis) and not receiving BoNT-A treatment. Nine respondents were caregivers of individuals living with spasticity who answered questions about BoNT-A experiences for the patient. The mean [95% CI] respondent age was 47.2 [45.9–48.5] years old and 53% of respondents were male (Table 1). Overall, 43% of respondents had spasticity due to stroke, 30% due to TBI, and 27% due to SCI. The mean [95% CI] age of onset of the neurological event was 42.7 [41.3–44.2] years old and the mean time since event was 4.6 [3.8–5.4] years. Respondents with post-stroke spasticity were older at the time of event than those with TBI and SCI (mean age of 45.3 vs. 40.6 and 40.8 years old, respectively). This age distribution indicates a younger population with stroke responded to the survey compared to incidence data (12). Across the etiologies, most respondents were employed (full or part-time).

***Results, Experiences of Symptom Re-emergence, Paragraph 1***

Symptom re-emergence between injections was common, with 83% of respondents saying they noticed their pre-existing symptoms reappearing between 2 injection sessions, and this was consistent across all etiologies (82–84%). The mean time to re-emergence of pre-existing symptoms was 89.4 [86.3, 92.4] days after BoNT-A injection, with 6% reporting symptom re-emergence within 2 months, 53% reporting within 2–3 months and 28% reporting symptom re-emergence only after >3 months. Overall, 22 respondents (13%) could not define the time to re-emergence of pre-existing symptoms. Of note, respondents receiving BoNT-A and concomitant oral medications experienced symptom re-emergence more frequently than those who only received BoNT-A injections and those who also received physiotherapy (89 vs. 81 and 79%, respectively).

***Results, Physician-Patient Communication About Symptom Re-emergence, Paragraph 1***

Most respondents (94%) said they had discussed the potential for symptom re-emergence between injections with their doctor. When asked whether they report symptom re-emergence to their physician, most (92%) said they inform their physician. The majority (66%) said they report their symptom re-emergence immediately, regardless of symptom intensity while 15% only reported symptom re-emergence if the symptoms were severe. Overall, 8% of respondents (*n* = 12) said they do not inform their doctor. Following a report of symptom re-emergence, the most common management approaches were to add adjunctive treatment (36%), increase the dose of BoNT-A (28%) and to wait for the next injection (27%). Another 26% reported they went back to their physician for an earlier than planned session.

***Discussion, Paragraph 5***

Our findings offer some important points for physician–patient discussion during treatment initiation and goal setting, monitoring and achievement. The data show that, from the patients' perspective, the effects of BoNT-A are not immediate, take time to reach their full potency, and do not always last throughout the time interval between two sessions. Given the high expectations many patients have of BoNT-A therapy (15), it is important that they understand the realistic objectives and the limitations of their treatment, and the probable time course of symptom relief. Injectors can only adjust the treatment regimen if they have adequate information at hand, and this will often depend on the patient being able to communicate how and when they re-experience their symptoms, as well as what is the maximum level of severity they consider acceptable just before the next injection. Our results show that many patients are able to describe the time course of effects in a consistent way. In this survey only 8% of respondents did not inform their doctor of the reappearance of their symptoms between two sessions of injections. This number was lower than we expected, and may reflect the population sample which is relatively young, with good cognitive function, and a proactive approach to disease management (as evidenced by joining a social media group for people living with chronic conditions). Patients with cognitive dysfunction or a lesser functional expectation to their condition may require some prompting questions to tease out these points. Indeed, 13% of respondents in this survey could not readily define the usual time taken to symptom reemergence. As such, it may also be helpful to develop educational visual tools (app based or diaries) explaining what to expect from BoNT-A treatment and when, allowing patients or their caregivers to record their experience, including waning of effect.

The authors apologize for these errors and state that they do not change the scientific conclusions of the article in any way. The original article has been updated.

