# Supplementary material for: Patient Perspectives on the Therapeutic Profile of Botulinum Neurotoxin Type A in Spasticity
Source: Front Neurol. 2020 May 7;11:388. doi: 10.3389/fneur.2020.00388 (PMC7233119; doi:10.3389/fneur.2020.00388)
Supplement: Supplementary file 1 [file Data_Sheet_1.PDF]

Understanding the waning of Botulinum Toxin Type A effects and its impact on the quality of life of patients living with spasticity

Description message:

Hello,

We invite you to participate in an international survey designed **to better understand patients' and caregivers' experience with Botulinum Toxin Type A injections**. These injections are given to improve symptoms related with spasticity, which could include muscle spasms, rigidity and pain, unwanted movement of the affected limb, difficulty using the arm or/and the leg…

1. What is the aim of this survey?

Botulinum toxin effect usually peaks around 4 to 6 weeks after injection, then it slowly decreases. This decrease is called the waning of botulinum toxin effect. The aim of this survey is to better understand patients' experience with botulinum toxin type A injections, in particular:

- to better understand how patients experience the waning of botulinum toxin type A effects
- to identify the impact of the waning of botulinum toxin type A effects on the patient's quality of life
- to describe patient populations profile
- To assess the reasons for stopping botulinum toxin type A injections. Do you have to participate?

Your participation in this survey is entirely voluntary. It is your choice whether to participate or not. If you agree to participate, you will be asked to click on the "Start" button below. Please note that you will not receive any compensation or remuneration for your participation in this survey.

You have the right to refuse to participate or to withdraw participation at any time for any reason. This will not impact the standard of care or medical services you currently receive or will receive in the future.

3. What will happen if you do not want to participate?

If you decide not to participate in this survey, the quality of care or medical services you receive or will receive will not be impacted in any way.

4. What Personal Data will be collected in relation to this survey?

Else Care SAS, publisher of Carenity website, may collect Personal Data when you answer the survey, including connection data (User ID, login, start and end time of survey), profile data (gender, year of birth, country of residence), health data and medical profile (pathology, prior symptoms and diagnosis, symptoms, treatment...) and impact of the disease on professional and personal life ("Personal Data").

5. What happens with your data?

No Personal Data collected through this survey will be transmitted, sold, shared or otherwise distributed to a third party.

Your Personal Data will be used to conduct this survey, conduct the analysis, reporting and regulatory submission, as the case may be, meet the above-mentioned goals of the survey, as well as for statistical purposes. We will not use your Personal Data for other purposes.

Information related to you will only be accessed by Else Care SAS. Only individuals at Else Care SAS with tangible and relevant need to see your information will have access to it.

The data collected through this survey will be consolidated anonymously and in aggregate form, and thus do not allow any personal identification of the participants.

These aggregate results will be shared with Ipsen, a pharmaceutical company that manufactures a symptomatic treatment for spasticity (Please note that details of product or indication availabilities vary from country to country).

This survey will be also the subject of communications during scientific congresses or scientific publications that will be redacted in collaboration with an international team of medical experts.

Your Personal Data are stocked by Else Care SAS until the study survey results have been published, after which your Personal Data will remain archived for the period of 5 years before being completely deleted.

Your Personal Data will be stored on secure servers and maintained strictly confidential.

6. What rights do you have regarding your Personal Data?

According to the current legislation, you have the right to:

- access and rectify your Personal Data;
- object to and delete your Personal Data;
- withdraw your consent at any time;
- request restriction of processing;
- be forgotten and to require the erasure of your Personal Data;
- the portability of your Personal Data;
- lodge a complaint with the CNIL.

To exercise your rights, you may, if you are logged into your Carenity account, send a private message to your Community manager by clicking here.

You may also contact the Else Care SAS' Data Protection Officer for any questions regarding your personal data, at the following e-mail address: dpo@carenity.com.

7. Within what framework are your data collected?

The treatment of your Personal Data is conducted based on your explicit consent, which is formalized by the click on the “Start” button below. You may withdraw your consent at any time without providing a reason, without affecting the lawfulness of the processing carried out prior to the withdrawal of your consent. This will not impact the standard of care or medical services you currently receive or will receive in the future.

8. Who is responsible for the treatment of my data?

IPSEN INNOVATION SAS, residing at 5 Avenue du Canada, Zone Industrielle de Courtaboeuf, Les Ulis, 91940, France, ("IPSEN") is the data controller with respect to the use of your Personal Data collected as part of this survey. The data controller may be contacted at the following email address: dataprivacy@ipsen.com.

9. Adverse Event Reporting

We are being asked by the law to pass on to IPSEN the details of adverse events and/or product complaints related to IPSEN's product that are raised during the course of the survey. Your information will of course, be treated confidentially. Should an adverse event and/or product complaint related to IPSEN's product arise during the survey, we will need to report this even if you have already reported it directly to the pharmaceutical company. In case of the need of further information regarding this adverse event, Else Care SAS will contact you, unless you disagree by contacting Else Care SAS' as mentioned above.

The above information on the way your data will be treated and about the terms of exercise of your rights are also in our Information Notice.

Your participation in the survey is voluntary. By clicking on the "Start" button, you confirm:
1. your willingness to participate in the survey;
2. that you give your explicit consent to have your personal data collected and processed in the way described above and in our Information Notice.

You may withdraw your consent at any time without providing a reason, without affecting the lawfulness of the processing carried out prior to the withdrawal of your consent.

Thank you in advance for your contribution,

The Carenity Team

Black: to all respondents or to patients only
Orange: to caregivers only

During the questionnaire, you will be asked **the name of *the last Botulinum Toxin Type A injections you received***. *If you do not remember, or are not sure about the name, please refer to the doctor or the medical centre before starting the survey.*

This questionnaire should take you 10 to 15 minutes to complete.
Once you have finished answering a question, you can click on the "Next" button to go to the next question. Please note that you will not be able to move back to the previous question after that.

Thank you for your participation.

A. Respondent's profile

1. **You are a:**

   (Single answer)

   ◻ Man

   ◻ Woman

2. **Your year of birth:**

   (yyyy) **[SCREEN OUT if age<18 y/o]**

3. **Your country of residence:**

   (Single answer)

   ◻ France

   ◻ Germany

   ◻ Italy

   ◻ Spain

   ◻ UK (England, Wales, Scotland, Northern Ireland)

   ◻ USA

   ◻ Other *[Specify]* **[SCREEN OUT]**

4. **Your profile:**

   (Single answer)

   ◻ Patient who had a stroke

   ◻ Caregiver of a patient who had a stroke

   ◻ Patient with a traumatic brain injury

   ◻ Caregiver of a patient with a traumatic brain injury

   ◻ Patient with a spinal cord injury

   ◻ Caregiver of a patient with a spinal cord injury

   ◻ None of the above **[SCREEN OUT]**

[Message to be displayed for the patients:]

INSERT:

In view of the questionnaire length we would like to ensure that you stay focused all through the questionnaire, we slipped three extra questions into the survey to test your attention. Don't be surprised!

5. **Is the patient you care for registered on Carenity?**

   (Single answer)

   ◻ Yes **> *[What is the username of the patient you care for?]***

   ◻ No

6. **Are you answering the questionnaire with the patient?**

   (Single answer)

   ◻ Yes

- ☐ No

7. **What is your relationship with the patient?**
   (Single answer)
   - ☐ The patient is my partner
   - ☐ The patient is my child
   - ☐ The patient is my mother/my father
   - ☐ The patient is my brother/sister
   - ☐ The patient is another member of my family
   - ☐ The patient is my friend
   - ☐ I am an employed helper
   - ☐ Other *[Specify]*

8. **The patient you care for is a:**
   (Single answer)
   - ☐ Man
   - ☐ Woman

9. **What is the year of birth of the patient you care for?**
   (yyyy) **[SCREEN OUT if age<18 y/o]**

10. **On average, how often do you take care of the patient?**
    (Single answer)
    - ☐ Less than once a week **[SCREEN OUT]**
    - ☐ At least 1 day a week
    - ☐ At least 2 days a week
    - ☐ At least 4 days a week
    - ☐ Everyday

11. **When did you start taking care of the patient?**
    (yyyy)
    - ☐ I do not remember

12. **Please tell us about your employment status.**
    **Full-time: more than 30 hours per week*
    ***Part-time: less than 30 hours per week*
    (Single answer)
    - ☐ I work full-time*
    - ☐ I work part-time** because of my condition
    - ☐ I work part-time** but it is not due to my condition
    - ☐ I do not work because of my condition
    - ☐ I do not work but it is not due to my condition (retired, …)
    - ☐ I am a full time student
    - ☐ Other *[Specify]*

12bis. Please tell us about the employment status of the patient you care for.

**Full time: more than 30 hours per week*

***Part-time: less than 30 hours per week*

(Single answer)

- ◻ He/she works full-time*
- ◻ He/she works part-time** because of his/her condition
- ◻ He/she works part-time** but it is not due to his/her condition
- ◻ He/she does not work because of his/her condition
- ◻ He/she does not work but it is not due to his/her condition (retired, …)
- ◻ He/she is a full time student
- ◻ Other [Specify]

[Message to be displayed for the caregivers who answered yes to question 6:]

INSERT:

In the following part of the questionnaire, the questions are intended for patients, please answer for the patient.

[The caregivers who answered yes to question 6 will be redirected to the question 13 of the patient's questionnaire]

13. When did your stroke/traumatic brain injury/spinal cord injury occur?

13bis. When did the patient's stroke/traumatic brain injury/spinal cord injury occur?

(yyyy)

- ◻ I do not remember

Let's see how focus you are!

1/3. Please check every number higher than 5.

(Several possible answers)

- ◻ 1
- ◻ 2
- ◻ 4
- ◻ 7
- ◻ 8

14. During the past 12 months, at the worst time, which of the following symptoms have you experienced?

(Several possible answers)

- ◻ Muscle spasms
- ◻ Muscle stiffness/rigidity (including painful cramps)
- ◻ Muscle pain
- ◻ Unwanted movement of the affected limb
- ◻ Difficulties moving my leg, falling, tripping, loss of balance
- ◻ Difficulties moving my arm/hand, extending my arm, opening my hand
- ◻ None of the above **[SCREEN OUT]**

14bis. During the past 12 months, at the worst time, which of the following symptoms has the patient experienced?

(Several possible answers)

- ☐ Muscle spasms
- ☐ Muscle stiffness/rigidity (including painful cramps)
- ☐ Muscle pain
- ☐ Unwanted movement of the affected limb
- ☐ Difficulties moving his/her leg, falling, tripping, loss of balance
- ☐ Difficulties moving his/her arm/hand, extending his/her arm, opening his/her hand
- ☐ None of the above **[SCREEN OUT]**

15. During the past 12 months, at the worst time, which of the following aspects of your daily life have been impacted as a consequence of your stroke/traumatic brain injury/spinal cord injury?

(Several possible answers, randomised)

- ☐ Grooming myself
- ☐ Dressing myself
- ☐ Washing myself
- ☐ Preparing my meal
- ☐ Moving around alone
- ☐ Grabbing, reaching, releasing objects
- ☐ Lack of sleep
- ☐ Fatigue
- ☐ Self-confidence
- ☐ Leisure
- ☐ Relationship with family and friends
- ☐ Ability/desire to socialize (approach others)
- ☐ Willingness to perform activities
- ☐ Depression and mood alterations (sadness, agitation, irritability…)
- ☐ Work
- ☐ Sexual life
- ☐ Anxiety about the future
- ☐ Other *[Specify]*
- ☐ None

15bis. During the past 12 months, at the worst time, which of the following aspects of the patient's daily life have been impacted as a consequence of his/her stroke/traumatic brain injury/spinal cord injury?

(Several possible answers, randomised)

- ☐ Ability to groom him/herself
- ☐ Ability to dress him/herself
- ☐ Ability to wash him/herself
- ☐ Ability to prepare their meal
- ☐ Ability to move around alone
- ☐ Grabbing, reaching, releasing objects
- ☐ Lack of sleep
- ☐ Fatigue
- ☐ Self-confidence

- ◻ Leisure
- ◻ Relationship with family and friends
- ◻ Ability/desire to socialize (approach others)
- ◻ Willingness to perform activities
- ◻ Depression and mood alterations (sadness, agitation, irritability…)
- ◻ Work
- ◻ Sexual life
- ◻ Anxiety about the future
- ◻ Other *[Specify]*
- ◻ None

INSERT:

In the following questions, we will use the word **"spasticity"** to refer to the symptoms you selected in the previous question among **muscle spasms, muscle stiffness/rigidity, muscle pain, unwanted movement of the affected limb, difficulties using the leg** (i.e. falling, tripping, loss of balance), **or difficulties using the arm/hand** (extending the arm, opening the hand…)**.**

[In the following questions, we will ask the respondents about their current and past treatments. Patients who have not received Botulinum Toxin Type A injections will be screened out (Q16 to Q17).]

16. Which treatments are you currently receiving for your spasticity?

Spasticity refers to one or several of the following symptoms: muscle spasms, muscle stiffness/rigidity, muscle pain, unwanted movement of the affected limb, difficulties using the leg (i.e. falling, tripping, loss of balance) or difficulties using the arm (extending the arm, opening the hand…).

(Several possible answers)

- ◻ Oral (tablet or liquid) medication (muscle relaxant, oral baclofen…)
- ◻ Botulinum toxin type A injections (injections into the muscle)
- ◻ Botulinum toxin type B injections (injections into the muscle)
- ◻ Phenol injections
- ◻ Alcohol injections
- ◻ Intrathecal baclofen/other intrathecal medication therapy
- ◻ Physiotherapy at home
- ◻ Physiotherapy at hospital/clinic or in private practice
- ◻ Home based self-rehabilitation
- ◻ Surgery
- ◻ Traditional/complementary medicine (e.g. Chinese medicine, osteopathy, homeopathy, etc)
- ◻ Other *[Specify]*
- ◻ I don't take any specific treatment for my spasticity

16bis. Which treatments is the patient currently receiving for his/her spasticity?

Spasticity refers to one or several of the following symptoms: muscle spasms, muscle stiffness/rigidity, muscle pain, unwanted movement of the affected limb, difficulties using the leg (i.e. falling, tripping, loss of balance) or difficulties using the arm (extending the arm, opening the hand…).

(Several possible answers)

- ◻ Oral (tablet or liquid) medication (muscle relaxant, oral baclofen…)
- ◻ Botulinum toxin type A injections (injections into the muscle)
- ◻ Botulinum toxin type B injections (injections into the muscle)
- ◻ Phenol injections
- ◻ Alcohol injections
- ◻ Intrathecal baclofen/other intrathecal medication therapy

- ◻ Physiotherapy at home
- ◻ Physiotherapy at hospital/clinic or in private practice
- ◻ Home based self-rehabilitation
- ◻ Surgery
- ◻ Traditional/complementary medicine (e.g. Chinese medicine, osteopathy, homeopathy, etc)
- ◻ Other *[Specify]*
- ◻ He/she does not take any specific treatment for his/her spasticity

17. In the past, which treatments did you receive for your spasticity?

Spasticity refers to one or several of the following symptoms: muscle spasms, muscle stiffness/rigidity, muscle pain, unwanted movement of the affected limb, difficulties using the leg (i.e. falling, tripping, loss of balance) or difficulties using the arm (extending the arm, opening the hand…).

(Several possible answers)

- ◻ Oral (tablet or liquid) medication (muscle relaxant, oral baclofen…)
- ◻ Botulinum toxin type A injections (injections into the muscle)
- ◻ Botulinum toxin type B injections (injections into the muscle)
- ◻ Phenol injections
- ◻ Alcohol injections
- ◻ Intrathecal baclofen/other intrathecal medication therapy
- ◻ Physiotherapy at home
- ◻ Physiotherapy at hospital/clinic or in private practice
- ◻ Home based self-rehabilitation
- ◻ Surgery
- ◻ Traditional/complementary medicine (e.g. Chinese medicine, osteopathy, homeopathy, etc)
- ◻ Other *[Specify]*
- ◻ I did not take any specific treatment for my spasticity in the past

17bis. In the past, which treatments did the patient receive for his/her spasticity?

Spasticity refers to one or several of the following symptoms: muscle spasms, muscle stiffness/rigidity, muscle pain, unwanted movement of the affected limb, difficulties using the leg (i.e. falling, tripping, loss of balance) or difficulties using the arm (extending the arm, opening the hand…).

(Several possible answers)

- ◻ Oral (tablet or liquid) medication (muscle relaxant, oral baclofen…)
- ◻ Botulinum toxin type A injections (injections into the muscle)
- ◻ Botulinum toxin type B injections (injections into the muscle)
- ◻ Phenol injections
- ◻ Alcohol injections
- ◻ Intrathecal baclofen/other intrathecal medication therapy
- ◻ Physiotherapy at home
- ◻ Physiotherapy at hospital/clinic or in private practice
- ◻ Home based self-rehabilitation
- ◻ Surgery
- ◻ Traditional/complementary medicine (e.g. Chinese medicine, osteopathy, homeopathy, etc)
- ◻ Other *[Specify]*
- ◻ He/she did not take any specific treatment for his/her spasticity in the past

[Respondents who have not received Botulinum Toxin Type A injections are screened out (Q16 to Q17).]

[For patients/ caregivers of patients who received Botulinum Toxin Type A injections in the past only]

18. Did you stop receiving Botulinum Toxin Type A injections more than one year ago?
18bis. Did the patient stop receiving Botulinum Toxin Type A injections more than one year ago?
(Single answer)

- ◻ Yes **[SCREEN OUT]**
- ◻ No

B. Experience with Botulinum Toxin Type A injections

INSERT for patient who received Botulinum Toxin Type A in the past only:
For the next part of the questionnaire, we are going to ask you questions about your Botulinum Toxin Type A injections. **Please answer for the period of time you were receiving Botulinum Toxin Type A injections**.

INSERT for caregivers of patient who received Botulinum Toxin Type A in the past only:
For the next part of the questionnaire, we are going to ask you questions about patient's Botulinum Toxin Type A injections. **Please answer for the period of time he/she was receiving Botulinum Toxin Type A injections.**

19. What is the name of the Botulinum Toxin Type A injections you last received?
19bis. What is the name of the Botulinum Toxin Type A injections the patient last received?
If you don't remember the name of the last Botulinum Toxin Type A injections received, please refer to the doctor or the medical centre.
(Single answer)
- □ I know the name *[Specify]*
- □ I do not know

20. In which limbs do you receive Botulinum Toxin Type A injections for your spasticity symptoms?
20bis. In which limbs does the patient receive Botulinum Toxin Type A injections for his/her spasticity symptoms?
Spasticity refers to one or several of the following symptoms: muscle spasms, muscle stiffness/rigidity, muscle pain, unwanted movement/posture of the affected limb, difficulties using the leg (i.e. falling, tripping, loss of balance) or difficulties using the arm (extending the arm, opening the hand…).
(Several possible answers)
- □ Right arm
- □ Left arm
- □ Right leg
- □ Left leg
- □ Other *[Specify]*

21. When did you start receiving Botulinum Toxin Type A injections?
21bis. When did the patient start receiving Botulinum Toxin Type A injections?
(yyyy)
- □ I do not remember

22. Have you received more than one session of Botulinum Toxin Type A injections?
(Single answer)
- □ Yes, I have received several sessions of Botulinum Toxin Type A injections
- □ No, I have only received Botulinum Toxin Type A injections once **[SCREEN OUT]**

22bis. Has the patient received more than one session of Botulinum Toxin Type A injections?
(Single answer)
- ◻ Yes, he/she has received several sessions of Botulinum Toxin Type A injections
- ◻ No, he/she has only received Botulinum Toxin Type A injections once **[SCREEN OUT]**

23. In general, how many times per year do you receive Botulinum Toxin Type A injections?
23bis. In general, how many times per year does the patient receive Botulinum Toxin Type A injections?
(Minimum=1, maximum=6)
(Numeric)
- ◻ I do not know

24. How many months ago did you receive your last Botulinum Toxin Type A injections for your spasticity?
24bis. How many months ago did the patient receive his/her last Botulinum Toxin Type A injections for his/her spasticity?
Spasticity refers to one or several of the following symptoms: muscle spasms, muscle stiffness/rigidity, muscle pain, unwanted movement of the affected limb, difficulties using the leg (i.e. falling, tripping, loss of balance) or difficulties using the arm (extending the arm, opening the hand…).
(Single answer)
- ◻ Less than 1 month ago
- ◻ 1 month to less than 2 months ago
- ◻ 2 months to less than 3 months ago
- ◻ 3 months to less than 4 months ago
- ◻ 4 months to less than 6 months ago
- ◻ 6 months ago or more
- ◻ I do not remember

25. What was the time between your two last sessions of Botulinum Toxin Type A injections for your spasticity?
25bis. What was the time between his/her two last sessions of Botulinum Toxin Type A injections for his/her spasticity?
Spasticity refers to one or several of the following symptoms: muscle spasms, muscle stiffness/rigidity, muscle pain, unwanted movement of the affected limb, difficulties using the leg (i.e. falling, tripping, loss of balance) or difficulties using the arm (extending the arm, opening the hand…).
(Single answer)
- ◻ Less than 3 months
- ◻ 3 months to less than 4 months
- ◻ 4 months to less than 6 months
- ◻ 6 months or more
- ◻ I do not remember

26. Please select the answer which best describes how your sessions of Botulinum Toxin Type A injections are scheduled.
(Single answer)
- ◻ The interval between two sessions of injections is always the same.
- ◻ The interval between two sessions of injections is not regular, the sessions are arranged when needed depending on my symptoms.
- ◻ The interval between two sessions of injections is not regular, the sessions are arranged depending on the availability of the doctor/hospital.

- □ The interval between two sessions of injections is not regular, the sessions are arranged depending on when it is possible for me to go to the hospital.
- □ Other [*Specify]*

26bis. What do you think about the way your sessions of Botulinum Toxin Type A are scheduled?

(Single answer)

- □ It's well adapted to my situation.
- □ I would like the sessions to be arranged differently.
- □ Other [*Specify]*

26ter. Please select the answer which best describes how the patient's sessions of Botulinum Toxin Type A Injections are scheduled.

(Single answer)

- □ The interval between two sessions of injections is always the same.
- □ The interval between two sessions of injections is not regular, the sessions are arranged when needed depending on his/her symptoms.
- □ The interval between two sessions of injections is not regular, the sessions are arranged depending on the availability of the doctor/hospital.
- □ The interval between two sessions of injections is not regular, the sessions are arranged depending on when it is possible for him/her to go to the hospital.
- □ Other [Specify*]*

26qua. What do you think about the way the patient's sessions of Botulinum Toxin Type A are scheduled?

(Single answer)

- □ It's well adapted to his/her situation.
- □ He/She would like the sessions to be arranged differently.
- □ Other [*Specify]*

27. In general, how many days or weeks after your Botulinum Toxin Type A injections do you experience the first effect of the treatment on your spasticity symptoms?

Spasticity refers to one or several of the following symptoms: muscle spasms, muscle stiffness/rigidity, muscle pain, unwanted movement of the stiff limb, difficulties using the leg (i.e. falling, tripping, loss of balance) or difficulties using the arm (extending the arms, opening the hand…).

(Numeric, minimum=0)

[The first effect of the treatment on your spasticity symptoms] (in days or weeks) □ I do not know

27bis. In general, how many weeks or months after your Botulinum Toxin Type A injections do you experience the maximum effects of the treatment on your spasticity symptoms?

Spasticity refers to one or several of the following symptoms: muscle spasms, muscle stiffness/rigidity, muscle pain, unwanted movement of the stiff limb, difficulties using the leg (i.e. falling, tripping, loss of balance) or difficulties using the arm (extending the arms, opening the hand…).

(Numeric, minimum=0)

[The maximum effects of the treatment on your spasticity symptoms] (in weeks or months) □ I do not know

27ter. In general, how many days or weeks after his/her Botulinum Toxin Type A injections does the patient experience the first effect of the treatment on his/her spasticity symptoms?

Spasticity refers to one or several of the following symptoms: muscle spasms, muscle stiffness/rigidity, muscle pain, unwanted movement of the stiff limb, difficulties using the leg (i.e. falling, tripping, loss of balance) or difficulties using the arm (extending the arm, opening the hand…).

(Numeric, minimum=0)

[The first effect of the treatment on his/her spasticity symptoms] (in days or weeks) □ I do not know

27qua. In general, how many weeks or months after his/her Botulinum Toxin Type A injections does the patient experience the maximum effect of the treatment on his/her spasticity symptoms?

Spasticity refers to one or several of the following symptoms: muscle spasms, muscle stiffness/rigidity, muscle pain, unwanted movement of the stiff limb, difficulties using the leg (i.e. falling, tripping, loss of balance) or difficulties using the arm (extending the arm, opening the hand…).

(Numeric, minimum=0)

[The maximum effects of the treatment on his/her spasticity symptoms] (in weeks or months) □ I do not know

Let's see how focus you are!

2/3. Please check every number higher than 5.

(Several possible answers)

- □ 7
- □ 2
- □ 8
- □ 4
- □ 1

C. Experience with the waning of Botulinum Toxin Type A effects

28. During your first sessions of Botulinum Toxin Type A injections, did your doctor inform you that symptoms could return between two sessions of injections?

28bis. During the first sessions of Botulinum Toxin Type A injections, did the doctor of the patient inform him/her that symptoms could return between two sessions of injections?

(Single answer)

- □ Yes
- □ No
- □ I don't remember

29. In general, do your spasticity pre-existing symptoms reappear between 2 sessions of Botulinum Toxin Type A injections?

29bis. In general, do his/her spasticity pre-existing symptoms reappear between 2 sessions of Botulinum Toxin Type A injections?

Spasticity refers to one or several of the following symptoms: muscle spasms, muscle stiffness/rigidity, muscle pain, unwanted movement of the affected limb, difficulties using the leg (i.e. falling, tripping, loss of balance) or difficulties using the arm (extending the arm, opening the hand…).

(Single answer)

- □ Yes
- □ No **[Respondents who checked "no" and are still receiving Botulinum Toxin Type A treatment will be sent to the end of the questionnaire]**

30. In general, how long after your Botulinum Toxin Type A injections does your spasticity pre-existing symptoms begin to reappear?

30bis. In general, how long after his/her Botulinum Toxin Type A injections does the patient's spasticity pre-existing symptoms begin to reappear?

Spasticity refers to one or several of the following symptoms: muscle spasms, muscle stiffness/rigidity, muscle pain, unwanted movement of the affected limb, difficulties using the leg (i.e. falling, tripping, loss of balance) or difficulties using the arm (extending the arm, opening the hand…).

(Numeric, minimum=0)

(In weeks or months) □ I do not know

31. Please select the pre-existing symptoms which reappear between two sessions of Botulinum Toxin Type A injections in their order of reappearance:

The first symptom you select is the one which reappears first and so on. If a symptom does not appear between two Botulinum Toxin Type A treatments, please do not select it. If you want to change the order, or remove a symptom from the ranking, you can unselect it by clicking on it again. 1: this symptom reappears first, 2: this symptom is the second to reappear…

(Ranking question, minimum 1 item)

- □ Muscle spasms
- □ Muscle stiffness/rigidity (including painful cramps)
- □ Muscle pain
- □ Unwanted movement of the affected limb
- □ Difficulties moving my leg, falling, tripping, loss of balance
- □ Difficulties moving my arm/hand, extending the arm, opening the hand

31bis. Please select the pre-existing symptoms which reappear between two sessions of Botulinum Toxin Type A injections in their order of reappearance:

The first symptom you select is the one which reappears first and so on. If a symptom does not appear between two Botulinum Toxin Type A treatments, please do not select it. If you want to change the order, or remove a symptom from the ranking, you can unselect it by clicking on it again. 1: this symptom reappears first, 2: this symptom is the second to reappear…

(Ranking question, minimum 1 item)

- □ Muscle spasms
- □ Muscle stiffness/rigidity (including painful cramps)
- □ Muscle pain
- □ Unwanted movement of the affected limb
- □ Difficulties moving his/her leg, falling, tripping, loss of balance
- □ Difficulties moving his/her arm/hand, extending the arm, opening the hand

INSERT:
In the following question(s), we will ask you about the intensity of the symptoms at 3 moments of the treatment with Botulinum Toxin Type A:

- *When the effects of the injections are at their maximum (peak effect)*
- *When the pre-existing symptoms reappear/ the effects of the injections start wearing off*
- *The day before receiving new injections*

[Questions 32 will be asked for each symptom selected at question 31 or 31bis]

32. How would you rate the intensity of your [SYMPTOMS Q31] at these 3 moments?
32bis. How would you rate the intensity of the patient's [SYMPTOMS Q31] at these 3 moments?
Spasticity refers to one or several of the following symptoms: muscle spasms, muscle stiffness/rigidity, muscle pain, unwanted movement of the affected limb, difficulties using the leg (i.e. falling, tripping, loss of balance) or difficulties using the arm (extending the arm, opening the hand…).
For each aspect, drag the slider to the desired position: left end = no symptoms, right end = very strong symptoms.

When the effects of the injections are at their maximum (peak effect)	No symptoms -------------O------------ Very strong symptoms
When the pre-existing symptoms reappear/ the effects of the injections start wearing off	No symptoms -------------O------------ Very strong symptoms
The day before receiving new injections	No symptoms -------------O------------ Very strong symptoms

Let's see how focus you are!
3/3. Please check every number higher than 5.
(Several possible answers)

- ☐ *1*
- ☐ 8
- ☐ 4
- ☐ 2
- ☐ 7

D. Impact of the waning of Botulinum Toxin Type A effects on the patient's quality of life and expectations

[Q33 and Q33bis are asked for patients currently working only]

33. **How does the reappearance of your spasticity pre-existing symptoms between two sessions of Botulinum Toxin Type A injections affect your work?**
Spasticity refers to one or several of the following symptoms: muscle spasms, muscle stiffness/rigidity, muscle pain, unwanted movement of the affected limb, difficulties using the leg (i.e. falling, tripping, loss of balance) or difficulties using the arm (extending the arm, opening the hand…).
(Several possible answers)
- ◻ I have to take time off work
- ◻ I do not feel comfortable at work
- ◻ I have to work less (start later and/or stop earlier than I would otherwise, …)
- ◻ I have to work at home
- ◻ I am not as efficient at work as I usually am
- ◻ Other *[Specify]*
- ◻ It does not affect my work

33bis. How does the reappearance of his/her spasticity pre-existing symptoms between two sessions of Botulinum Toxin Type A injections affect his/her work?
Spasticity refers to one or several of the following symptoms: muscle spasms, muscle stiffness/rigidity, muscle pain, unwanted movement of the affected limb, difficulties using the leg (i.e. falling, tripping, loss of balance) or difficulties using the arm (extending the arm, opening the hand…).
(Several possible answers)
- ◻ He/she has to take time off work
- ◻ He/she does not feel comfortable at work
- ◻ He/she has to work less (start later and/or stop earlier than he/she would otherwise, …)
- ◻ He/she has to work at home
- ◻ He/she is not as effective at work as he/she usually is
- ◻ Other *[Specify]*
- ◻ It does not affect his/her work

[Q34 is only asked for caregivers currently working]

34. **As a caregiver, does the reappearance of the patient's pre-existing symptoms between two sessions of Botulinum Toxin Type A injections affect your work life?**
(Single answer)
- ◻ Yes, I have to change my work schedule to take care of the patient during these periods
- ◻ Yes, I have to stop working to take care of the patient during these periods
- ◻ No

[Q35 to Q35bis will be asked for the five following items:]

- **Ability to perform daily tasks (e.g. grooming, dressing…)**
- **Ability to move around**
- **Lack of sleep/fatigue**
- **Self-confidence**
- **Relationship with family and friends**

35. How would you rate the impact of your spasticity on your [QUALITY_OF_LIFE_ITEM] at these 3 moments?

35bis. How would you rate the impact of the patient's spasticity on his/her [QUALITY_OF_LIFE_ITEM] at these 3 moments?

Spasticity refers to one or several of the following symptoms: muscle spasms, muscle stiffness/rigidity, muscle pain, unwanted movement of the affected limb, difficulties using the leg (i.e. falling, tripping, loss of balance) or difficulties using the arm (extending the arm, opening the hand…).

For each aspect, drag the slider to the desired position: left end = no impacts, right end = very strong impacts.

When the effects of the injections are at their maximum (peak effect)	No impact -------------O------------ Very strong impact
When the pre-existing symptoms reappear/ the effects of the injections start wearing off	No impact -------------O------------ Very strong impact
The day before receiving new injections	No impact -------------O------------ Very strong impact

36. When your spasticity symptoms reappear between two sessions of Botulinum Toxin Type A injections, do you inform your doctor about it?

Spasticity refers to one or several of the following symptoms: muscle spasms, muscle stiffness/rigidity, muscle pain, unwanted movement of the affected limb, difficulties using the leg (i.e. falling, tripping, loss of balance) or difficulties using the arm (extending the arm, opening the hand…).

(Several possible answers)

- ☐ Yes, I inform my doctor immediately regardless of the intensity of my symptoms
- ☐ Yes, I inform my doctor immediately but only when my symptoms are severe
- ☐ Yes, I inform my doctor at the next appointment regardless of the intensity of my symptoms
- ☐ Yes, I inform my doctor at the next appointment but only when my symptoms are severe
- ☐ Yes, I inform my doctor to schedule a new appointment
- ☐ Other *[Specify]*
- ☐ No **> Q38**

36bis. When his/her spasticity symptoms reappear between two sessions of Botulinum Toxin Type A injections, do you or does he/she inform(s) the doctor about it?
Spasticity refers to one or several of the following symptoms: muscle spasms, muscle stiffness/rigidity, muscle pain, unwanted movement of the affected limb, difficulties using the leg (i.e. falling, tripping, loss of balance) or difficulties using the arm (extending the arm, opening the hand…).
(Several possible answers)

- ◻ Yes, immediately regardless of the intensity of the symptoms
- ◻ Yes, immediately but only when the symptoms are severe
- ◻ Yes, at the next appointment regardless of the intensity of the symptoms
- ◻ Yes, at the next appointment but only when the symptoms are severe
- ◻ Yes, we inform his/her doctor to schedule a new appointment
- ◻ Other *[Specify]*
- ◻ No **> Q38**

[Q37 and Q37bis are only asked for patients and caregivers of patients who have informed their doctor about the reappearance of their pre-existing symptoms]

37. How did your doctor react when you informed him/her about the earlier reappearance of your spasticity symptoms?
Spasticity refers to one or several of the following symptoms: muscle spasms, muscle stiffness/rigidity, muscle pain, unwanted movement of the affected limb, difficulties using the leg (i.e. falling, tripping, loss of balance) or difficulties using the arm (extending the arm, opening the hand…).
(Several possible answers)

- ◻ My doctor prescribed an additional treatment (e.g. oral treatment/alcohol or phenol injection/physiotherapy) to combat the symptoms
- ◻ My doctor increased the dosage of the Botulinum Toxin Type A injection used in the next treatment session
- ◻ My doctor switched my Botulinum Toxin Type A to another brand
- ◻ My doctor asked me to come earlier for the next Botulinum Toxin Type A injection
- ◻ My doctor told me I have to wait for the next injection
- ◻ Yes, I inform my doctor to schedule a new appointment
- ◻ Other *[Specify]*
- ◻ I do not know

37bis. How did his/her doctor react when he/she informed him/her about the earlier reappearance of his/her spasticity symptoms?
Spasticity refers to one or several of the following symptoms: muscle spasms, muscle stiffness/rigidity, muscle pain, unwanted movement of the affected limb, difficulties using the leg (i.e. falling, tripping, loss of balance) or difficulties using the arm (extending the arm, opening the hand…).
(Several possible answers)

- ◻ His/her doctor prescribed an additional treatment (e.g. oral treatment/alcohol or phenol injection/physiotherapy) to combat the symptoms
- ◻ His/her doctor increased the dosage of the Botulinum Toxin Type A injection used in the next treatment session
- ◻ His/her doctor switched his/her Botulinum Toxin Type A to another brand
- ◻ His/her doctor asked him/her to come earlier for the next Botulinum Toxin Type A injection
- ◻ His/her doctor told him/her he/she has to wait for the next injection
- ◻ Yes, he/she informs his/her doctor to schedule a new appointment
- ◻ Other *[Specify]*

- ◻ I do not know

[Q38 and Q38bis are only asked for patients or caregivers who have not informed their doctor about the reappearance of the pre-existing symptoms]

38. Why did you not inform your doctor about the earlier reappearance of your spasticity symptoms?
Spasticity refers to one or several of the following symptoms: muscle spasms, muscle stiffness/rigidity, muscle pain, unwanted movement of the affected limb, difficulties using the leg (i.e. falling, tripping, loss of balance) or difficulties using the arm (extending the arm, opening the hand…).
(Several possible answers)

- ◻ I did not think my doctor could do anything about that
- ◻ I did not want to disturb my doctor
- ◻ I did not have time to inform my doctor
- ◻ My doctor did not have the time
- ◻ I was worried that my doctor would prescribe an additional treatment (e.g. oral treatment/ alcohol or phenol injection)
- ◻ I did not want to be prescribed Botulinum Toxin Type A injections more frequently
- ◻ I did not want to have more muscles injected during the same injection session
- ◻ I did not want to be prescribed a different Botulinum Toxin treatment
- ◻ I was worried that my doctor would not believe me
- ◻ Other *[Specify]*
- ◻ I do not know

38bis. Why did he/she not inform his/her doctor about the earlier reappearance of his/her spasticity symptoms?
Spasticity refers to one or several of the following symptoms: muscle spasms, muscle stiffness/rigidity, muscle pain, unwanted movement of the affected limb, difficulties using the leg (i.e. falling, tripping, loss of balance) or difficulties using the arm (extending the arm, opening the hand…).
(Several possible answers)

- ◻ He/she did not think his/her doctor could do anything about that
- ◻ He/she did not want to disturb his/her doctor
- ◻ He/she did not have time to inform his/her doctor
- ◻ His/her doctor did not have the time
- ◻ He/she was worried that his/her doctor would prescribe an additional treatment (e.g. oral treatment/ alcohol or phenol injection)
- ◻ He/she did not want to be prescribed Botulinum Toxin Type A injections more frequently
- ◻ He/she did not want to have more muscles injected during the same injection session
- ◻ He/she did not want to be prescribed a different Botulinum Toxin treatment
- ◻ He/she was worried that his/her doctor would not believe him/her.
- ◻ Other *[Specify]*
- ◻ I do not know

[Only for patient:]

39. What improvements with your Botulinum Toxin Type A treatment do you want in order to avoid reappearance of symptoms between sessions of injections?
(Single answer)

- ◻ Have a treatment with benefits lasting longer
- ◻ Have more muscles injected
- ◻ Have higher doses injected
- ◻ The possibility to have more frequent injections
- ◻ Other *[Specify]*
- ◻ None

E. Reasons for stopping Botulinum Toxin Type A injections

[For patients and caregivers of patients who have stopped Botulinum Toxin Type A injections only]

40. Why did you stop receiving Botulinum Toxin Type A injections for your spasticity?
Spasticity refers to one or several of the following symptoms: muscle spasms, muscle stiffness/rigidity, muscle pain, unwanted movement of the affected limb, difficulties using the leg (i.e. falling, tripping, loss of balance) or difficulties using the arm (extending the arm, opening the hand…).
(Several possible answers)

- ◻ I had side effect(s) *[Specify]*
- ◻ The treatment did not work well enough
- ◻ My doctor told me to stop the injections
- ◻ The treatment was too expensive
- ◻ Logistics constraints (travel to hospital, time spent on injections…) were too inconvenient
- ◻ I wanted to take less medication
- ◻ Injections were too painful
- ◻ Another treatment was proposed as an alternative to Botulinum Toxin injections (e.g. surgery)
- ◻ I did not need it anymore
- ◻ Other *[Specify]*
- ◻ I don't remember

40bis. Why did the patient stop receiving Botulinum Toxin Type A injections for his/her spasticity?
Spasticity refers to one or several of the following symptoms: muscle spasms, muscle stiffness/rigidity, muscle pain, unwanted movement of the affected limb, difficulties using the leg (i.e. falling, tripping, loss of balance) or difficulties using the arm (extending the arm, opening the hand…).
(Several possible answers)

- ◻ He/she had side effect(s) [*Specify*]
- ◻ The treatment did not work well enough
- ◻ His/her doctor told him/her to stop the injections
- ◻ The treatment was too expensive
- ◻ Logistics constraints (travel to hospital, time spent on injections…) were too inconvenient
- ◻ He/she wanted to take less medication
- ◻ Injections were too painful
- ◻ Another treatment was proposed as an alternative to Botulinum Toxin injections (e.g. surgery)
- ◻ He/she did not need it anymore
- ◻ Other *[Specify]*
- ◻ I don't remember
